# Alterations of sympathetic dynamics after atrial fibrillation ablation by analysis sympathetic nerve activity provide prognostic value for recurrence and mechanistic insights into ablation

**DOI:** 10.3389/fcvm.2022.1024156

**Published:** 2022-11-30

**Authors:** Jien-Jiun Chen, Chen Lin, Yuan-Cheng Chuang, Shu-Fang Lee, Tse-Yu Lin, Chieh-Cheh Yu, Chia-Ti Tsai, Min-Tsun Liao, Tin-Tse Lin, Lian-Yu Lin, Men-Tzung Lo

**Affiliations:** ^1^Division of Cardiology, Department of Internal Medicine, Yunlin Branch of National Taiwan University Hospital, Douliu, Taiwan; ^2^Department of Biomedical Sciences and Engineering, National Central University, Taoyuan, Taiwan; ^3^Division of Cardiology, Department of Internal Medicine, College of Medicine, National Taiwan University and Hospital, Taipei, Taiwan; ^4^Division of Cardiology, Department of Internal Medicine, Hsinchu Branch of National Taiwan University Hospital, Hsinchu, Taiwan

**Keywords:** skin sympathetic nerve activity, neuECG, atrial fibrillation, catheter ablation, cryoablation

## Abstract

**Background:**

Pulmonary vein isolation (PVI) is the cornerstone of atrial fibrillation (AF) ablation. Success is associated with autonomic function modulation; however, the relationship between the changes after ablation is not fully understood. We aimed to investigate the effect of ablation on autonomic modulation by skin sympathetic nerve activity (SKNA) using conventional electrocardiogram (ECG) electrodes and to predict the treatment success.

**Methods:**

We enrolled 79 patients. We recorded neuECG for 10 min at 10 kHz before and after ablation. The NeuECG was bandpass-filtered (500–1,000 Hz) and integrated at intervals of 100 ms (iSKNA). iSKNA was averaged over different time windows (1-, 5-,10-s; aSKNAs), and burst analyses were derived from aSKNAs to quantify the dynamics of sympathetic activities. AF recurrence after 3 months was defined as the study endpoint.

**Results:**

Sixteen patients experienced AF recurrence after the ablation. For burst analysis of 1-s aSKNA, the recurrence group had a higher bursting frequency than the non-recurrence group (0.074 ± 0.055 vs. 0.109 ± 0.067; *p* < 0.05) before ablation. The differences between pre- and post-ablation of firing duration longer than 2 s were more in the non-recurrence group (2.75 ± 6.41 vs. −1.41 ± 5.14; *p* < 0.05), while no significant changes were observed in the percentage of duration longer than 10 s using 5-s aSKNA. In addition, decreases in differences in firing frequency and percentage of both overall firing duration and longer firing duration (> 2 s) between pre- and post-ablation were independently associated with AF recurrence and more area under receiver operating characteristics (ROC) curve in combination with CHADS_2_ score (0.833).

**Conclusion:**

We demonstrated the applicability of neuECG for determining sympathetic modulation during AF ablation. Decreasing sympathetic activity is the key to successful ablation.

## Introduction

Catheter ablation for paroxysmal or persistent atrial fibrillation (AF) has been regarded as a therapeutic choice, especially for patients who are refractory to one or two types of anti-arrhythmia drugs. Isolation of the pulmonary vein by radiofrequency catheter ablation (RFCA) or cryoballoon ablation has been reported with satisfactory results, with a recurrence rate of approximately 10–20%. Several methods have been reported to predict recurrence. Reports have shown a correlation between sympathetic nervous system activation and the onset and termination of AF (1). Some methods for AF ablation also target the parasympathetic ganglion plexus and have been shown to have a high success rates (2, 3). However, its correlation with sympathetic activity has not yet been discussed in detail. A study of heart rate variability tests for AF has been proposed (4, 5). However, this method is not applicable in patients with persistent AF, and in some patients with paroxysmal AF, sinus node dysfunction coexists, making the accuracy of this method questionable.

Recent studies have shown that signals with frequencies beyond 150 Hz on electrocardiography (ECG), usually considered as noise, can convey information about skin sympathetic nerve activity (SKNA) (1, 6). Although the method has been validated by invasive nerve recording showing that SKNA is consistent with stellate ganglion and thoracic sympathetic nerve activities (6, 7), the associations between the changes in the amplitude or frequency of SKNA firing and autonomic nervous system (ANS) control are still controversial. For example, the clustered firing of integrated SKNA (iSKNA; the sum of the activity over 100 ms) and elevated average SKNA (aSKNA; an average of iSKNA over 10 s) were found to be associated with both the initiation and termination of cardiac arrhythmias, such as AF and ventricular tachycardia (1, 8). Therefore, several short-term SKNA bursting analyses have been proposed to better represent the underlying sympathetic activity during different disease states, such as arrhythmogenicity in patients with coronary heart disease and changes in autonomic function in patients with acute myocardial infarction and critical illness (9–11).

In this study, we believe that short-term burst analysis of SKNA detected by neuECG could provide information on sympathetic control in patients with AF. We collected data from patients who underwent RFCA or cryoablation and recorded their SKNA. By analyzing the complex dynamics of SKNA and its clinical outcomes, we hypothesized that substrate modification could influence the short-term bursting characteristics of SKNA and that short-term sympathetic activities could carry information related to the maintenance mechanism of AF and could potentially be used as indicators for predicting AF recurrence.

## Materials and methods

From November 2017 to January 2020, we recruited patients who underwent RFCA or cryoablation for AF for the first time. The choice of treatment method depended on the attending electrophysiologist and the patient’s preferences. For RFCA, we performed only pulmonary vein isolation (PVI), and the technical details have been described in our previous publication (12). For cryoablation, we used standard methods and confirmed entry and exit blocks by electro anatomical mapping using the Ensite Presidion mapping system (Abbott, USA) (if patients are still in AF rhythm after ablation, we check the entry block only). In addition to routine continuous ECG monitoring, an additional channel of surface electrocardiogram was recorded for 10 min digitized at 10,000 Hz by a fully programmable amplifier/data acquisition unit (MP36, BIOPAC, USA) for the analysis of SKNA. Before ablation, we recorded the SKNA for 10 min, after propofol-conscious sedation and before vessel cannulation. After ablation, we re-recorded SKNA in patients who were still in a sedated state to avoid the possible influence of the environment. We did our best to maintain the same sedation level perceived by our trained staff and used a similar dosage of propofol. We converted the AF to sinus rhythm and checked the entry and exit blocks. The patients’ AF types, CHADS_2_ (congestive heart failure, hypertension, age, diabetes, stroke), CHA_2_DS_2_VASc scores (congestive heart failure, hypertension, diabetes, stroke, vascular disease, sex) (13), left ventricle function, and left atrium size were reviewed using charts. The patients were followed up for recurrence with a blanking period of 3 months (14, 15). Recurrence was defined as documented AF on 12-lead ECG or 24-h Holter ECG (13, 16, 17). We continued to follow up with the patients until June 2021 and recorded if there was a recurrence of AF. The study did not involve the clinical management of the patients. Offline analysis of the SKNA data was performed using custom-developed MATLAB programs. This study was performed in compliance with the principles of the Declaration of Helsinki. This study was approved by the local ethics committee for analysis (202107096RINA).

### Data processing

**FIGURE 1:**
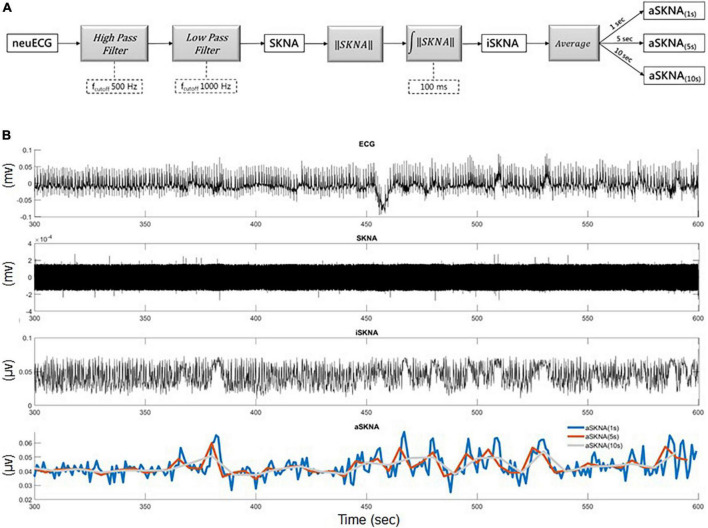
**(A)** The framework for skin nerve activity (SKNA) extraction: neuECG signal was originally recorded for 10 min sampling at 10,000 Hz and the neural activity signal was extracted by a 500-Hz high-pass filter and then a 1,000 Hz low-pass filter to remove the ECG signal. **(B)** Illustration of the extracted iSKNA (integrated SKNA) and aSKNA (averaged SKNA) signals: (1) the recorded neuECG signal; (2) the SKNA signal after high-pass filtering; (3) the iSKNA signal was derived by rectifying and summing up the SKNA signal with a non-overlapping 100 ms time window; (4) the iSKNA signal can be further averaged for different durations (e.g., 1, 5, or 10 s) to represent the dynamics of the sympathetic system interacting with the parasympathetic system for different time frames.

The electrical signal on the skin was detected using standard ECG patch electrodes connected to a fully programmable amplifier (MP36, BIOPAC, USA). A high-pass filter with a cut-off frequency of 500 Hz was used to distinguish the electrical signals from different sources, such as electrocardiograms and myopotential signals. In addition, a 1,000-Hz low-pass filter was used to attenuate the electrical noise by radio frequency interference (Figure 1A). The filtered signals, namely, SKNA, were rectified. The summation of the instantaneous rectified SKNA over a 100-ms time window is denoted as integrated SKNA (iSKNA). We analyzed the average iSKNA for each time window (1, 2, and 10 s). The averaged iSKNA of the 1-/5-s recordings were used as a parameter to represent overall SKNA (Figure 1B). Deriving ideas from frequency domain analysis in heart rate variability tests (18), we assumed that a 1-s time window was more likely to represent a balance between the sympathetic and parasympathetic activity, while windows larger than 5 s might represent sympathetic activities more. Windows > 10 s were not applied for further analysis because of the limited recording time.

### Burst analysis

**FIGURE 2:**
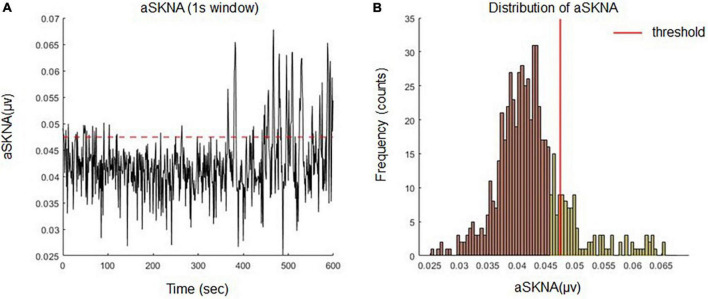
**(A)** Definition of burst activities of aSKNA signal by an individualized threshold. **(B)** The amplitudes of the aSKNA signal were classified into two clusters (background noise and sympathetic nerve activity) by K means algorithm. The cutoff threshold of each subject was determined by the mean value plus three times the standard deviation of the background group and the burst activity can be viewed as the activities over background noise.

The presence of SKNA bursts may be related to the initiation and termination of cardiac arrhythmia (8). Burst activity can be differentiated from the baseline, which consists of random single spikes with large amplitudes and durations. To differentiate between baseline and burst (7, 8), we used the K-means algorithm to cluster unsupervised data (aSKNA) into two groups (background noise and SKNA) (Figure 2B). The cutoff threshold of each subject was determined by the mean value plus three times the standard deviation of the background noise group, and the burst activities can be viewed as activities over background noise (Figure 2A). A complete burst starts at the point where the amplitude of an aSKNA signal exceeds the threshold and ends at the point where the amplitude of the signal is below the threshold. The aSKNA parameters were calculated as follows:

1. The burst amplitude (μV) is the average voltage during a burst within the evaluated time frame.
2. Burst frequency (bursts/min) is the total number of “On,” amplitudes of aSKNA that exceed the threshold for every minute.
3. Burst duration (%) = total span of burst (min)/total span of the time frame being evaluated (min) × 100.
4. The study duration was further divided into short and long periods. The long firing duration in 1-s window mean > 2 s and that in 5-s window implies > 10 s.
5. The total burst area (μV⋅min) is the sum of the burst areas (μV min) within the time frame being evaluated.

### Statistical analysis

Continuous variables with normal distribution were expressed as mean ± standard deviation. Categorical variables were expressed as percentages. The demographics, comorbid diseases, and medication use of the surviving and expired patients were compared. The normality of the variables was tested using the Shapiro–Wilk test, the between-group differences of continuous variables with normal distribution were tested using Student’s *t*-test, and the Mann–Whitney *U* test was used for the non-normally distributed variables. Categorical variables were compared between the patients with different outcomes using Fisher’s exact test. The hazard ratios of variables with significant between-group differences were further estimated using the Cox proportional hazard regression model. The optimal dichotomization cut-off points of the variables were determined by the best results of the log-rank test sought from the 30th to the 70th percentile by the fifth percentile increment for each time. The multivariate model was adjusted for age, sex, treatment, and other comorbidities. Kaplan–Meier survival and log-rank analyses were performed to test whether the event-free probabilities of the stratified groups were significantly different. In addition, the area under the receiver operating characteristic curves (AUC) of the SKNA parameters and CHADS_2_ score were computed to evaluate the discriminative ability, and the model incorporating the proposed parameters was also compared using the bootstrap method. All statistical analyses were performed using R software (Version 3.5.0). Statistical significance was set at *p*-value < 0.05.

## Results

A total of 79 patients were enrolled in the study. General patient characteristics are listed in Table 1. Sixteen of the 79 patients had recurrent AF after ablation. Diabetes was more prevalent in the recurrence group (*p* = 0.045) and patients with recurrence had higher CHADS_2_ scores. The remaining parameters are listed in Table 2. Echocardiographic measurements of left atrium size and left ventricular relaxation in both groups were similar.

**TABLE 1:** Baseline characteristics of the study participants.

Basic characteristics	No recurrent	Recurrence	*P*-value
	*N* = 63	*N* = 16	
Age (year)	64 (15)	66 (16.75)	0.291
Male, no. (%)	39 (61.9)	13 (81.3)	0.121
Paroxysmal atrial fibrillation, no. (%)	50 (79.4)	10 (62.5)	0.140
History of heart failure	9 (14.3)	3 (18.8)	0.456
Hypertension	36 (57.1)	12 (75.0)	0.154
Diabetes mellitus	12 (19.0)	7 (43.8)	**0.045**
Prior stroke/TIA	5 (7.9)	3 (18.8)	0.200
Peripheral artery disease	8 (12.7)	4 (25.0)	0.197
Coronary artery disease	6 (9.5)	3 (18.8)	0.261
CHADS_2_ score	1 (2)	2 (2)	0.012
CHA_2_DS_2_VASc score	2 (2)	2.5 (3.5)	0.081
Cryoablation	43 (68.3)	8 (50.0)	0.143

Values are expressed as mean ± standard deviation, median (interquartile range), or number (percentage).

TIA, transient ischemic attack; CHADS_2_, congestive heart failure, hypertension, age, diabetes, stroke; CHA_2_DS_2_VASc, congestive heart failure, hypertension, diabetes, stroke, vascular disease, sex.

Bold values mean *p* < 0.05.

**TABLE 2:** Relevant data.

	No recurrent	Recurrence	*P*-value
	*N* = 63	*N* = 16	
Pre-HR (63 vs. 16)	66 (26)	73.5 (23.5)	0.550
Post-HR (63 vs. 16)	70.0 ± 11.8	71.3 ± 16.0	0.727
Delta HR (63 vs. 16)	−1.7 ± 16.1	−3.6 ± 15.7	0.680
LA diameter (40 vs. 14)	42.0 ± 5.6	44.1 ± 6.4	0.241
E (41 vs. 13)	84.5 ± 25.1	88.4 ± 25.3	0.640
E’ lateral (12 vs. 4)	10.05 (6.03)	7.75 (7.10)	0.671
E’ medial (12 vs. 3)	7.8 ± 2.8	8.3 ± 5.6	0.824
LVEF (39 vs. 13)	68.4 ± 10.0	64.4 ± 8.0	0.175

Values are expressed as the mean ± standard deviation, median (interquartile range), or number (percentage).

HR, heart rate; LA, left atrium; LVEF, left ventricular ejection fraction.

### Changes of short-term sympathetic nerve activity after catheter ablation

The burst frequency of 1-s SKNA was significantly increased after ablation (0.08 ± 0.058 vs. 0.098 ± 0.062 bursts/min; *p* = 0.03), and bursting patterns shifted toward increasing long-duration bursting (5.764 ± 5.092% vs. 7.709 ± 5.696%; *p* = 0.01), while the duration of bursting remained the same (13.65 ± 7.59% vs. 14.51 ± 6.97%; *p* = 0.33) (Table 1). However, there were no significant differences in the SKNA over 5 s.

### Differences between short-term sympathetic nerve activity in patients with and without recurrence

**FIGURE 3:**
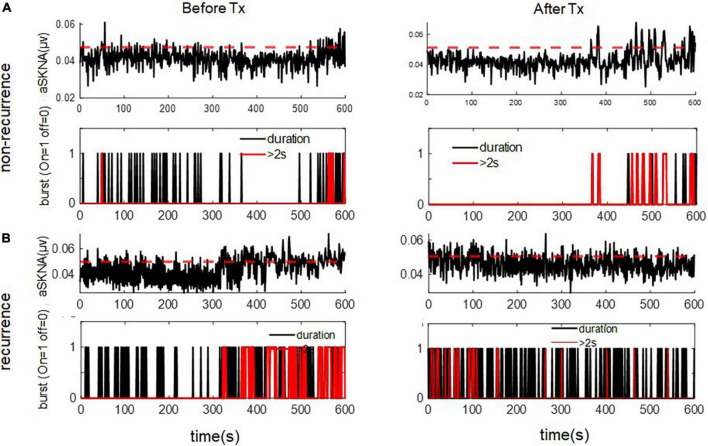
The representative figure showed firing duration in a 1-s window during baseline (Before Treatment) and after ablation (After Treatment) in **(A)** recurred and **(B)** non-recurred patients. The post-ablation burst duration, esp. long duration (> 2 s) was more frequent in the non-recurrence group. The upper panels of **(A,B)** represent the temporal changes of mean aSKNA (black line) and the determined threshold (red line). The lower panels of **(A,B)** showed the epochs with short duration of burst (black) and epochs with burst duration > 2 s (red).

**FIGURE 4:**
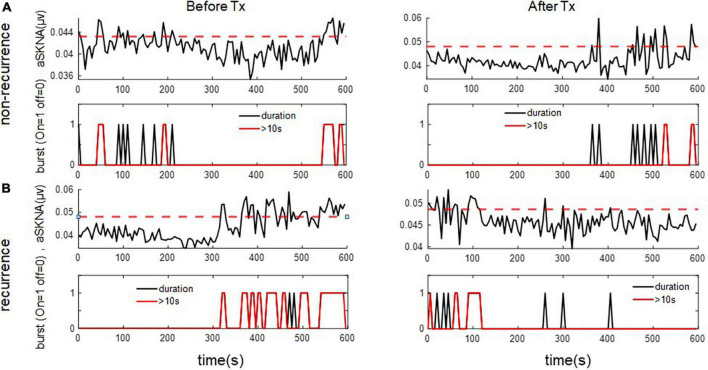
The representative figure showed firing duration in a 5-s window during baseline (Before Treatment) and after ablation (After Treatment) in **(A)** recurred and **(B)** non-recurred patients. The post-ablation burst duration, esp. long duration (> 10 s) was more frequent in the non-recurrence group. The upper panels of **(A,B)** represent the temporal changes of mean aSKNA (black line) and the determined threshold (red line). The lower panels of **(A,B)** showed the epochs with short duration of burst (black) and epochs with burst duration > 10 s (red).

For the 1-s SKNA (Table 3), the firing frequency before ablation was higher in the recurrent group (0.074 ± 0.055 vs. 0.109 ± 0.067 bursts/min, *p* = 0.036). After ablation, although the aSKNA parameters were similar, the changes (post-ablation minus pre-ablation) in the firing frequency and duration, especially for the percentage of long-duration epochs (i.e., > 2 s), were significant. Compared with the pre-ablation aSKNA, the recurrence group had a lower firing frequency and longer duration epochs. The aSKNA analyses of patients are shown in Figure 3. For the 5-s SKNA burst analysis, the firing frequency and long-duration epochs were significantly lower in the recurrence group (Table 4). For ablation changes, only the firing frequency was significantly decreased in the recurrent group. A representative patient is shown in Figure 4.

**TABLE 3:** SKNA (1 s).

SKNA	No recurrent	Recurrence	*P*-value
	*N* = 63	*N* = 16	
**Pre-ablation**			
Baseline (μV)	0.041 ± 0.013	0.040 ± 0.011	0.647
Threshold (μV)	0.051 ± 0.012	0.051 ± 0.016	0.855
Frequency (b/m)	0.074 ± 0.055	0.109 ± 0.067	**0.036**
Duration (%)	12.828 ± 6.910	16.979 ± 9.505	0.051
Duration, long (%)	5.286 ± 0.4.780	7.927 ± 5.961	0.065
Duration, short (%)	7.542 ± 5.203	9.052 ± 7.238	0.343
Amplitude (μV)	0.065 ± 0.043	0.065 ± 0.041	1.000
Area (μV*min)	0.020 ± 0.013	0.025 ± 0.015	0.155
**Post-ablation**			
Baseline (μV)	0.042 ± 0.013	0.040 ± 0.013	0.663
Threshold (μV)	0.051 ± 0.019	0.054 ± 0.025	0.533
Frequency (b/m)	0.102 ± 0.063	0.084 ± 0.058	0.292
Duration (%)	14.611 ± 6.621	14.146 ± 8.445	0.813
Duration, long (%)	8.034 ± 5.847	6.521 ± 5.033	0.346
Duration, short (%)	6.577 ± 4.235	7.635 ± 5.940	0.420
Amplitude (μV)	0.059 ± 0.042	0.082 ± 0.085	0.319
Area (μV*min)	0.019 ± 0.018	0.036 ± 0.047	0.167
**Difference**			
Baseline (μV)	0.0002 ± 0.0076	0.002 ± 0.0087	0.994
Threshold (μV)	−0.0007 ± 0.0151	0.0035 ± 0.0345	0.392
Frequency (b/m)	0.0375 ± 0.0694	−0.0250 ± 0.0556	**0.006**
Duration (%)	1.783 ± 7.509	−2.833 ± 8.093	**0.034**
Duration, long (%)	2.749 ± 6.410	−1.406 ± 5.143	**0.019**
Duration, short (%)	−0.966 ± 6.284	−1.427 ± 8.388	0.808
Amplitude (μV)	−0.0059 ± 0.0563	0.0166 ± 0.0762	0.189
Area (μV*min)	−0.0009 ± 0.0217	0.0110 ± 0.0501	0.366

All values are expressed as mean ± SD.

SKNA: skin sympathetic nerve activity, please refer to text for definition.

Bold values mean *p* < 0.05.

**TABLE 4:** SKNA data (5 s).

SKNA	No recurrent	Recurrence	*P*-value
	*N* = 63	*N* = 16	
**Pre-ablation**			
Baseline (μV)	0.042 ± 0.013	0.041 ± 0.011	0.663
Threshold (μV)	0.049 ± 0.012	0.049 ± 0.017	0.991
Frequency (b/m)	0.069 ± 0.044	0.066 ± 0.033	0.823
Duration (%)	14.775 ± 9.551	16.146 ± 12.663	0.634
Duration, long (%)	4.683 ± 3.975	4.010 ± 2.792	0.527
Duration, short (%)	10.093 ± 9.194	12.135 ± 11.887	0.458
Amplitude (μV)	0.059 ± 0.034	0.058 ± 0.034	0.901
Area (μV*min)	0.017 ± 0.014	0.019 ± 0.018	0.613
**Post-ablation**			
Baseline (μV)	0.043 ± 0.013	0.043 ± 0.013	0.944
Threshold (μV)	0.049 ± 0.018	0.050 ± 0.019	0.706
Frequency (b/m)	0.086 ± 0.051	0.053 ± 0.031	**0.003**
Duration (%)	14.127 ± 7.348	12.111 ± 7.721	0.347
Duration, long (%)	6.217 ± 4.610	3.722 ± 2.517	**0.007**
Duration, short (%)	7.910 ± 6.661	8.389 ± 6.542	0.802
Amplitude (μV)	0.056 ± 0.045	0.082 ± 0.087	0.114
Area (μV*min)	0.013 ± 0.017	0.021 ± 0.025	0.108
**Difference**			
Baseline (μV)	0.0008 ± 0.0078	0.0019 ± 0.0066	0.612
Threshold (μV)	0.0000 ± 0.0134	0.0015 ± 0.0069	0.661
Frequency (b/m)	0.0168 ± 0.0622	−0.0144 ± 0.0309	**0.008**
Duration (%)	−0.6481 ± 10.741	−4.611 ± 11.091	0.206
Duration, long (%)	1.534 ± 5.652	−0.444 ± 2.595	0.191
Duration, short (%)	−2.183 ± 10.540	−4.167 ± 10.852	0.517
Amplitude (μV)	−0.0029 ± 0.0526	0.0226 ± 0.0529	0.096
Area (μV*min)	−0.0046 ± 0.0227	0.012 ± 0.0299	0.412

All values are expressed as mean ± SD. SKNA, skin sympathetic nerve activity.

Bold values mean *p* < 0.05.

### Prognostic values of the parameters derived from short-term burst analysis

**FIGURE 5:**
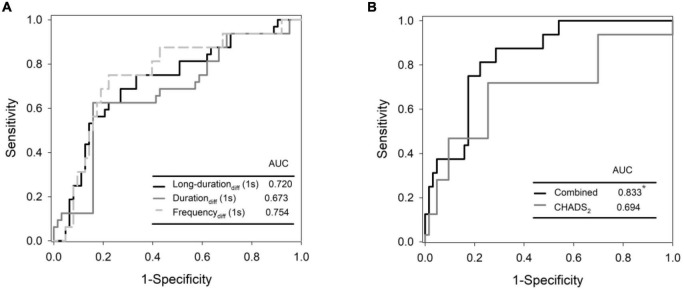
Receiver operating characteristic (ROC) curves from logistic regression models with CHADS_2_ score, pre-ablation 1-s frequency of skin SKNA, and post-ablation 5-s frequency of skin SKNA predicting the 3-month recurrence of AF **(A)** and the derived differences between pre- and post- ablation of long-duration, bursting frequency, and duration in combination with CHADS_2_ can better predict recurrence **(B)**. *Denotes most significant.

Predictors of recurrent AF were selected from 1-s and 5-s burst analyses according to the *p*-value, and the univariable and multivariable logistic regression models were shown in Table 5. Diabetes mellitus (DM) and high CHADS_2_ scores predict recurrence after ablation. Baseline (pre-ablation) firing frequency on a 1-s scale, post-ablation changes in firing frequency and duration in 1-s scale and firing frequency on a 5-s scale predict recurrence. After adjustment, DM and pre-ablation firing frequency on a 1-s scale and post-ablation firing frequency on a 5-s scale were independent predictors of recurrence. We also performed a multivariate analysis adjusting for DM and high CHA_2_DS_2_VASc scores (≥ 3). The predictive value of firing frequency at the 1-s scale was still significant (Supplementary Table 1). Receiver operating characteristic (ROC) curves from logistic regression models with CHADS_2_ score, pre-ablation 1-s frequency of skin SKNA, and post-ablation 5-s frequency of skin SKNA predicting the 3-month recurrence of AF (Figure 5A) and the derived differences between pre- and post-ablation of long-duration, bursting frequency, and duration in combination with CHADS_2_ can better predict recurrence (Figure 5B).

**TABLE 5:** Predictors of recurrent atrial fibrillation in the univariable and multivariable logistic regression model (1 and 5 s).

	Univariable			Multivariable*		
	OR	95% CI	*P*-value	OR	95% CI	*P*-value
Age (year)	1.037	0.980–1.097	0.204	1.007	0.912–1.113	0.887
Male, no. (%)	0.375	0.097–1.453	0.156	1.139	0.166–7.841	0.894
pAfib, no. (%)	0.425	0.130–1.385	0.155			
History of HF	1.385	0.328–5.845	0.658			
Hypertension	2.250	0.653–7.750	0.199			
Diabetes mellitus	3.306	1.025–10.660	**0.045**	0.371	0.040–3.484	0.386
Prior stroke/TIA	2.677	0.567–12.645	0.214			
PAD	2.292	0.592–8.866	0.230			
CAD	2.192	0.484–9.936	0.309			
CHADS_2_ score	1.774	1.147–2.743	**0.010**	2.758	1.033–7.360	**0.043**
CHA_2_DS_2_Vasc	1.373	0.974–1.935	0.070			
Cryoablation	0.465	0.153–1.417	0.178			
**Pre-ablation (1 s)**						
Frequency > 0.042 (b/m)	1.610	0.493–6.293	**0.450**			
**Difference (1 s)**						
Frequency > -0.012 (b/m)	0.095	0.024–0.319	**< 10^–3^**	0.062	0.011–0.263	**< 10^–3^**
Duration > -1.67 (%)	0.204	0.061–0.636	**< 10^–2^**	0.175	0.042–0.627	**< 10^–2^**
Duration, long > -0.83 (%)	0.204	0.061–0.636	**< 10^–2^**	0.165	0.037–0.636	0.01
**Pre-ablation (5 s)**						
Duration > 3.33 (%)	0.503	0.157–1.575	0.236			
Post-ablation (5 s)						
Frequency > 0.05 (b/m)	0.493	0.155–1.589	**0.228**			
**Difference (5 s)**						
Frequency > -0.008 (b/m)	0.268	0.075–0.853	0.030	0.288	0.072–1.033	0.06
Duration, long > 0 (%)	0.468	0.141–1.457	0.195			

*Adjusted for age, sex, persistent atrial fibrillation, CHADS_2_ score, and cryoballoon use.

CI, confidence interval; OR, odds ratio; pAF, paroxysmal atrial fibrillation; HF, heart failure; PAD, peripheral artery disease; CAD, coronary artery disease.

Please refer to the text for definition of SKNA parameters.

Bold values mean *p* < 0.05.

## Discussion

In this study, we found that neuECG is a feasible tool for evaluating SKNA, particularly in patients with AF. The firing frequency and duration were significantly lower in the post-ablation aSKNA data of the recurrent group in the 1-s window, and the firing frequency significantly decreased in the recurrence group in the 5-s window. We first shortened the window of aSKNA to 1 and 5 s and performed short- and long-burst analyses to facilitate our understanding of SKNA and neuECG.

Autonomic nervous system activation plays a crucial role in AF initiation and termination. Modulation of the sympathetic ANS showed that suppression of sympathetic tone wounds remarkably reduced atrial vulnerability to AF induction and post-ablation AF recurrence (2, 19, 20). However, in young patients, AF might trigger vagus nerve activation, which is called vagal AF (21). In our study, the mean patient age was approximately 65 years. Besides standard PVI, which is based on the theory of PV-triggered AF, there are many strategies of ablation, such as ablation of complex fractionated electrogram, creation of root line and mitral line, and BOX ablation, etc. (22). However, current randomized studies showed no difference in recurrence rate as compared with PV isolation only (17). Ganglion plexi are positioned mostly within the epicardial fat pad and are composed of various ganglia and interconnected axons (19, 23). In the atrium, ganglionic plexi are mostly located in the posterior wall of the left atrium, and ablation of the ganglion plexi has been proposed as one of the strategies (3, 24, 25). However, there is a lack of evidence of superiority in terms of the recurrence of ganglion plexi ablation. One possibility is that the plexi were mostly located in the left atrium-PV junction; therefore, PV isolation could also modify vagal activity. Our study showed that an increased SKNA firing frequency, especially for long-duration epochs, was the key to successful ablation. The baseline firing frequencies in the 1-s window were higher in the recurrent group. In the 1-s window, the recurrence group had a decreased SKNA firing frequency after ablation, which might mean that in the recurrence group, the sympathetic nervous system was more deteriorated, the parasympathetic nervous system was less active, and the patients’ AF neuronal trigger remained. In the 5-s window, the phenomenon was less prominent, which might mean that only sympathetic involvement is not as important as the balance between the sympathetic and parasympathetic nervous systems.

Previous studies have shown that burst amplitude was significantly higher in patients with AF clustering than in those without AF clustering, whereas the frequency and duration of SKNA bursts were not significantly different. SKNA precedes the onset and offset of AF and large bursts are associated with AF termination (1, 8). In our study, as illustrated in Figure 3, in the 1-s time window, aSKNA firing duration > 2 s was significantly higher after ablation. These results echo the idea that large bursts of SKNA are the key mechanisms for successful AF ablation. A large burst of SKNA in the 1-s window might indicate parasympathetic and sympathetic activation to avoid bradycardia or hypotension. In the 5-s window, pre-ablation long bursts and a higher frequency of bursts were associated with non-recurrence, and ablation decreased the SKNA firing frequency in the non-recurrence group. This finding supports the notion that sympathetic activity is involved in AF initiation and termination. However, from the 1-s window data, we believe that the interplay between the sympathetic nervous system and parasympathetic nervous system is more important than that of the sympathetic nervous system alone.

We used RF and cryoablation for PV isolation according to the electrophysiologist or patient preference. However, neither univariate nor multivariate predictors of recurrence were superior. Vagal reactions (i.e., severe bradycardia or hypotension) during cryoablation-based PV isolation are common. Some authors have considered vagal reactions during PV cryoablation to be a marker of ANS modification (26). We believe that cryoablation has effects on the ANS, according to our SKNA analysis. Further studies are needed to determine the degree of ANS modification by energy source and its correlation with clinical outcomes.

A recent study has showed that SKNA increased on day 1 after ablation and returned to baseline after 3 months (27). Increased SKNA levels were associated with early and late recurrences. Our study had a different study design in which we recorded the SKNA when patients were in a sedated state, and by which we believed we could minimize the effect of the environment and patients’ nervousness before and after the procedure. We also performed meticulous analyses of SKNA data, including burst duration and burst frequency analyses, according to published protocols (7). We also used different time windows of aSKNA to analyze our data to further expand our understanding of SKNA and neuECG, besides as predictors of recurrence.

This study provides insights into AF and AF ablation mechanisms, that is, besides creating entry and exit blocks in the pulmonary vein, what else does the energy (either radiofrequency or cryoablation) do to the ANS, which is crucial in cardiac arrhythmia initiation and termination. The interplay between the sympathetic and parasympathetic nervous systems is complex, and both the stellate ganglion and vagal nerve contain components of the sympathetic and parasympathetic nervous systems, as proven by staining of tyrosine hydroxylase and choline acetyltransferase at both sites (19). Using the neuECG method, the sympathetic nervous system and the complex interplay of the ANS can be evaluated non-invasively, but the initiation and termination of AF is also associated with other factors, such as PV triggers and arteriopathy in addition to neuronal mechanisms (13, 16, 22, 28). In addition to ablation, treatments for diabetes, obesity, hypertension, and heart failure are crucial for preventing recurrence (13, 29).

In addition to predicting AF recurrence, there are new applications in neuECG, such as acting as markers of orthostatic hypotension and recurrent syncope (30, 31), predicting the occurrence of ventricular arrhythmia in acute coronary syndrome (32), and correlating with intradialytic weight gain in patients with end-stage renal disease (33). It has also been proposed as a marker of fitness (34) and to predict neurological recovery of patients who had cardiac arrest and are undergoing therapeutic hypothermia (35). An ambulatory electrogram monitor was developed to record the SKNA (36). We hope that by real-time analysis of SKNA dynamics during AF ablation, we could monitor the effect of autonomic modulation and reduce recurrence after RF or cryoablation.

We used propofol during the whole procedure and recorded SKNA when patients were in a sedated state. Previous study showed that propofol can significantly suppress neural activities. Liu et al. showed that for patients undergoing cardioversion, bolus propofol administration can significantly suppress average SKNA amplitude (from 1.11 ± 0.25 μV to 0.77 ± 0.15 μV; *P* = 0.001) and the prolonged effect after the procedure can be noticed (37). However, sedation was necessary for patients who underwent AF ablation. If the SKNA was recorded before and after full recovery from sedation, the changes of SKNA before and after ablation can be attributed to the differences of the psychological conditions (e.g., feeling relieved on completion of ablation). We Therefore, we evaluated the changes of SKNA before and after ablation under sedation. In addition, we applied burst analysis which can take the firing pattern of the neural activity into account rather than only the absolute voltage summation and use different time windows of aSKNA to quantify the patterns of neural firing, to further expand our understanding of SKNA and neuECG.

This study has several limitations. The number of cases were limited, and the ablation method (RF or cryoablation) was not randomized. The temporal pattern of AF, i.e., paroxysmal or persistent AF might affect the recurrence rate, however we did not separate them into different patient groups. However, as shown in Table 5, we adjusted for persistent AF as a predictor of recurrence. Cases of patients receiving RFCA or cryoablation were also limited. Therefore, we could not compare the sympathetic modulation effect of different energy sources. We followed up for recurrence only, but the exact timing of the first recurrence could not be traced because some patients did not have symptoms. The recording time was only 10 min owing to time constraints in the procedures. Third, we did not differentiate between patients with vagal- and adrenergic-mediated AF. However, most patients were aged more than 50 years.

In conclusion, we found that AF ablation could modify the patterns of firing frequencies and duration of the sympathetic nervous system using neuECG methods and that increased long-duration epochs incorporated with a minimal decrease in firing frequency predicted AF recurrence. In addition, proving the efficacy of these methods, this study provides insights into possible AF mechanisms in patients with recurrence and potential responders to AF ablation.

## Data availability statement

The raw data supporting the conclusions of this article will be made available by the authors, without undue reservation.

## Ethics statement

The studies involving human participants were reviewed and approved by the Research Ethics Committee of National Taiwan University Hospital (202107096RINA). The patients/participants provided their written informed consent to participate in this study.

## Author contributions

J-JC designed the study, recorded the SKNA, analyzed the data, and drafted the manuscript. CL designed the study and analyzed the SKNA data. Y-CC, S-FL, and T-YL analyzed the SKNA data. C-CY and C-TT performed the clinical cases. M-TLi and T-TL performed the statistical analyses. L-YL supervised the study and critically reviewed the manuscript. CL and M-TLo were responsible for the study and reviewed all the materials and the manuscript. All authors contributed to the article and approved the submitted version.
