# Supplementary material for: Alterations of sympathetic dynamics after atrial fibrillation ablation by analysis sympathetic nerve activity provide prognostic value for recurrence and mechanistic insights into ablation
Source: Front Cardiovasc Med. 2022 Nov 30;9:1024156. doi: 10.3389/fcvm.2022.1024156 (PMC9748148; doi:10.3389/fcvm.2022.1024156)
Supplement: Supplementary file 1 [file Table_1.DOCX]

Table S1. Multivariate Cox regression for the model with DM and high CHA_2_DS_2_VASc scores

|  |  | **Multivariable*** | | | | |
| --- | --- | --- | --- | --- | --- | --- |
|  | OR | 95% CI | | | *P value* |  |
| **Pre-ablation (1s)** |  |  |  |  | | |
| Frequency>0.042 (b/m) | 1.67 | | 0.49-6.78 | **0.43** | | |
| **Difference (1s)** |  |  |  |  | | |
| Frequency>-0.012 (b/m) | 0.063 | | 0.012-0.248 | **<10^-3^** | | |
| Duration>-1.67 (%) | 0.173 | | 0.046-0.474 | **<10^-2^** | | |
| Duration, long>-0.83 (%) | 0.166 | | 0.043-0.556 | **<10^-2^** | | |
| **Pre-ablation (5s)** |  |  |  |  | | |
| Duration>3.33 (%) |  |  |  |  | | |
| **Post-ablation (5s)** |  |  |  |  | | |
| Frequency>0.05 (b/m) | 0.229 | | 0.058-0.769 | **.02** | | |
| **Difference (5s)** |  |  |  |  | | |
| Frequency>-0.008 (b/m) | 0.227 | | 0.058-0.769 | .02 | | |
| Duration, long>0 (%) |  |  |  |  | | |

*Adjusted for DM, and CHA_2_DS_2_VASc scores ≥ 3

Abbreviation: DM: diabetes mellitus; CI, confidence interval; OR, odds ratio
